# Retraction statement: HBx‐mediated decrease of AIM2 contributes to hepatocellular carcinoma metastasis

**DOI:** 10.1002/1878-0261.13333

**Published:** 2022-12-02

The above article, published online on 05 June 2017 in Wiley Online Library (wileyonlinelibrary.com), has been retracted by agreement between the journal Editor in Chief, Kevin Ryan, FEBS Press and John Wiley and Sons Ltd. The retraction has been agreed due to the identification of several duplications and errors in Figures 3, 4 and 6 and supplementary figure 5. There were also duplications present in some of the raw data provided retrospectively to the journal. An institutional investigation concluded that there had been unintentional misplacement of images in the figures. Given the extent of identified issues, the Editors have decided to retract the article.
